# Emergency radiology demand in a touristic region: residence status as a driver of imaging use

**DOI:** 10.1007/s10140-026-02464-4

**Published:** 2026-04-17

**Authors:** Milena Calabrese, Gianluca Capone, Marco Parillo, Federica Vaccarino, Carlo Cosimo Quattrocchi

**Affiliations:** 1 Faculty of Medicine and Surgery, University of Verona – Strada Le Grazie, Verona, 8 - 37134 Italy; 2 Radiology, Multizonal Unit of Rovereto and Arco, ASUIT, Ospedale Santa Maria del Carmine di Rovereto - Corso Verona, 4, Rovereto, 38068 Italy; 3 Department of Economics and Management, University of Parma, Via Kennedy, Parma, 6 - 43125 Italy; 4 Centre for Medical Sciences - CISMed, University of Trento, Via S. Maria Maddalena, 1, Trento, 38122 Italy

**Keywords:** Diagnostic imaging, Emergency care, Tourism, Hospital personnel management, Patient care management, Retrospective study.

## Abstract

**Purpose:**

To assess whether residence status influences the use of radiological services in emergency departments (EDs) in a high-tourism region of Northern Italy.

**Methods:**

This retrospective study analyzes all ED visits in the Autonomous Province of Trento from January 1, 2018 to December 31, 2024. Patients were categorized as residents or non-residents based on municipality of residence. Demographic, clinical, and contextual variables were extracted from the ED database. Regression models (OLS, Poisson, negative binomial, and logistic) were applied to evaluate determinants of radiological examination volume and workload, and the impact of residence status, including interaction with triage priority codes.

**Results:**

The sample includes 738,864 patients who underwent at least one radiological examination (overall volume of 1,474,937 imaging examinations). Non-residents accounted for 18.2% of patients and 20% of all radiological examinations. On average, they underwent significantly more imaging per visit than residents (2.19 vs. 1.95), even after controlling for confounding variables. Differences were most pronounced in high-priority cases: non-residents with red triage codes received nearly four additional examinations. Skeletal imaging was particularly over-represented among non-residents, consistent with trauma-related visits during tourism. Logistic regression showed non-residents had 31.9% higher odds of undergoing disproportionately high imaging volumes.

**Conclusion:**

Residence status significantly influences diagnostic imaging use in emergency settings. Non-residents, particularly in severe clinical conditions, undergo more extensive radiological evaluation. This likely reflects diagnostic uncertainty and lack of prior medical history. Findings underscore the need for adaptive resource planning in touristic areas and call for further studies on clinical appropriateness and outcome impact.

**Graphical Abstract:**

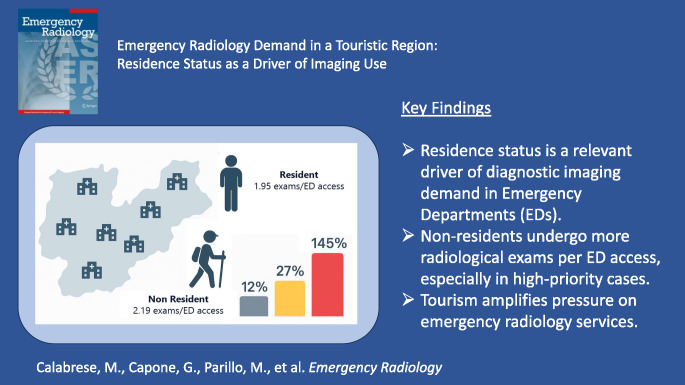

**Supplementary Information:**

The online version contains supplementary material available at 10.1007/s10140-026-02464-4.

## Introduction

Emergency departments (EDs) play a critical role in ensuring timely access to healthcare. This can be especially challenging in regions with significant fluctuations in population due to tourism. Several studies have highlighted how tourism alters the epidemiological and clinical profile of ED users [1, 2]. Tourists are often exposed to unfamiliar environments [3], climates, and physical activities, resulting in increased risks for trauma, infections, and exacerbations of pre-existing conditions [1, 2]. In particular, injuries among tourists are commonly associated with recreational activities [4], vehicle crashes, and pedestrian accidents [5], sometimes necessitating advanced imaging and prolonged ED stays [6]. These patterns of care often differ from those of resident patients and raise important concerns about the scalability and adaptability of emergency services in touristic regions [7, 8].

Imaging plays a central role in emergency diagnostics. Its use has expanded significantly over the past decades [9, 10], not only due to clinical advancements but also in response to evolving patterns of healthcare-seeking behavior and physicians’ reliance on radiology to reduce diagnostic uncertainty, generating a more than proportional increase in the radiologist workload [11]. Radiological examinations are resource-intensive, requiring specialized personnel and equipment, and their increased use among patients may reflect a combination of clinical complexity, precautionary practice in unfamiliar patients, and medico-legal considerations.

Despite these dynamics, there is limited empirical evidence comparing imaging use between residents and non-residents in EDs. Existing literature has primarily focused on broader indicators such as ED admission rates [1, 8], waiting times [6], or injury prevalence among tourists [2, 5], with less attention paid to diagnostic intensity. Furthermore, while demand forecasting for radiology services has been explored from a resource planning perspective [12–14], no study so far has linked such analyses to patient mobility or population dynamics in high-tourism areas. Given the economic reliance of some regions on tourism [15] and the complex perceptions of tourism’s impact among residents and service providers [3, 16], understanding how non-resident patients utilize emergency services is essential for planning, sustainability, and equity in healthcare delivery.

This study aims to fill that gap by examining radiological service utilization among ED patients in Trentino over a period of 7 years, comparing residents and non-residents. We aim to evaluate diagnostic intensity in terms of number and type of radiological examinations performed, adjusted for demographic, clinical, and temporal variables, eventually exploring the association between the residence status and the diagnostic pathways.

Our findings aim to support more informed policy and operational decisions in regions where healthcare infrastructure must flexibly accommodate seasonal and population-driven variation in demand.

## Materials and methods

### Study design

The study was conducted in accordance with the Declaration of Helsinki (as revised in 2013). This study is a quality care control investigation based on anonymized aggregated administrative data, and therefore did not require Institutional Review Board approval neither patients informed consent.

This study is based on data collected retrospectively from the 7 Hospitals with an active ED in the Autonomous Province of Trento, in Northern Italy, a renowned tourist destination both in winter and in summer. The sample for this study includes all patients who accessed an ED in the Province of Trento from 1 January 2018 to 31 December 2024 and that have undergone at least one radiological examination. Data for all patients and their radiological exams were extracted from the ED database using the management software Qlik­View (QlikTech, King of Prussia, PA, USA).

### Variables

The main variable of this study splits the sample into two study groups: patients who are residents in the province of Trento and non-resident patients. The dataset also included the following demographic, clinical, and ED access information: sex (male / female), age (including 7 categories: 0–14; 15–24; 25–34; 35–44; 45–54; 55–64; over 65), triage priority level (classified in 4 colors – white, green, yellow, red – until March 2021 and 5 colors – white, green, blue, orange, red – from April 2021; to simplify the analysis, we aggregated the codes merging the green and blue categories and the yellow and orange categories), motivation for ED access (including 7 categories: home, school or work accident; sport/skiing accident; road accident; non traumatic; trauma – accidental injury; trauma – violence of others; other), hospital ED where the access occurred, date and time of the access (classifying nighttime access as occurred between 8pm and 8am and weekend access as occurred on Saturday or Sunday). The imaging dataset included information about the type of the examination that was classified in 12 categories: ultrasound; subspecialty ultrasound; skeletal X-Rays; skeletal computed tomography (CT); abdomen X-Rays; abdomen CT; chest X-Rays; chest CT; head & neuro CT; angiography CT; other CT; other exams. By combining information from the two datasets, we built the following variables: the number of radiological examinations undertaken by each patient; the number of CT examinations undertaken by each patient; a binary variable that took the value of 1 if the patient underwent at least 1 CT scan, and 0 otherwise; a binary variable that took the value of 1 if the patient underwent at least 1 ultrasound, and 0 otherwise.

### Statistical and regression analysis

The analyses were conducted with the statistical software Stata 18 (StataCorp, College Station, TX, USA). Statistical analyses were conducted to compare across the two groups of resident and non-resident ED patients: (a) demographic characteristics (sex, age); (b) clinical information (priority triage code, motivation for access, type of imaging); (c) ED access details (hospital, year, weekday and day/night access); (d) the number and type of radiological examinations the patients undertook following their ED access. All these variables were expressed as percentages and total number of events and were analyzed using Chi-square tests, with Bonferroni adjustment to correct for multiple comparisons.

Regression analyses were employed to investigate the determinants of the number of examinations that each patient undertook. Ordinary Least Squares (OLS) provided a more direct interpretation of the results. Poisson regression considered the count nature of the dependent variables. Variance-to-Mean ratio and Pearson Chi-Square test were computed to test for overdispersion. To deal with it, a threefold strategy was adopted: (a) a negative binomial (NB) regression, that also estimates a dispersion parameter; (b) an OLS regression with the exclusion o f the most extreme (top 1%) cases in terms of number of examinations undertaken (Trimmed OLS); (c) a logistic regression (Logit) that predicted the probability of being in the top 5% of patients in terms of number of examinations undertaken. All models included the full set of demographic, clinical, and ED access control variables. All coefficients with a *p*-value < 0.05 were considered statistically significant. Standard errors were clustered at the hospital-year level to account for correlation in clinical practice patterns and policy changes (e.g., during the COVID-19 pandemic).

To address heterogeneity in examination complexity, we apply a twofold strategy. First, we run the regression analysis using just the number of CT examinations as dependent variable. Second, we derive a time-weighted imaging burden metric (Prestational Time, PT) using the Italian Society of Medical Radiology (SIRM) 2022 qualitative–quantitative appropriateness framework for diagnostic imaging [17, 18]. PT is expressed in minutes and represents a standardized, exam-level burden of the radiologic act, including, within a single reference value, the main components of the radiology workflow: clinical history review, justification of the examination, informed consent (when applicable), examination execution, and reporting. Importantly, PT does not represent the directly measured real-time duration of a specific examination in the emergency department for an individual patient, but rather a standardized reference value linked to the examination category within the SIRM framework. For each imaging examination, we assigned the corresponding PT value (in minutes) after mapping local examination codes/descriptions to SIRM examination categories. For each ED visit, the SIRM-based time-weighted imaging burden was calculated as the sum of PT values across all imaging examinations performed during that visit.

## Results

### Baseline differences between resident and non-resident patients

During the study period (2018–2024), 738,864 patients accessed the EDs of one of the hospitals of the Province of Trento undertaking at least one radiological examination. The residents in the Province of Trento represented 81.8% (*N* = 604,163) of the total, whereas the non-resident were 18.2% (*N* = 134,701).

Table 1 presents the demographic, clinical, and access characteristics of ED patients, highlighting the differences between residents and non-residents. Non-residents were more likely to be male (54.8% vs. 51.7%, *p* < 0.01) and younger across all age groups except those aged 65 and older. The most marked difference was found in the 25–34 age group (12.3% vs. 7.3%, *p* < 0.01). There were small but statistically significant differences in triage priority codes between residents and non-residents. Non-residents were overrepresented in the blue/green code (70.3% vs. 68.1%, *p* < 0.01), whereas a higher percentage of residents had a white (4.6% vs. 4.9%, *p* < 0.01), yellow/orange (22.9% vs. 23.9%, *p* < 0.01) or red classification (2.2% vs. 3.2%, *p* < 0.01). Larger differences were observed in the motivation for the ED access: non-residents were over-represented in the sport/skiing accident (23.1% vs. 3.4%, *p* < 0.01), accidental injury trauma (31.1% vs. 25.2%, *p* < 0.01), and road accident (4.2% vs. 3.2%, *p* < 0.01) categories, and underrepresented in the non-traumatic (36.2% vs. 57.0%, *p* < 0.01) and home, school or work accident (4.4% vs. 10.2%, *p* < 0.01) categories.

**Table 1:** Demographic, clinical, and access characteristics of ED patients, highlighting the differences between residents and non-residents

	Non Residents		Residents		*p*-value
	Freq	Percent	Freq	Percent	
Number of patients	134,701		604,163		
Sex					
Female	60,942	45.24	291,840	48.30	< 0.001
Male	73,759	54.76	312,323	51.70	< 0.001
Age Category					
0–14	12,665	9.40	49,887	8.26	< 0.001
15–24	17,340	12.87	50,432	8.35	< 0.001
25–34	16,617	12.34	44,334	7.34	< 0.001
35–44	15,578	11.56	50,590	8.37	< 0.001
45–54	20,382	15.13	71,232	11.79	< 0.001
55–64	18,478	13.72	78,513	13.00	< 0.001
65+	33,641	24.97	259,175	42.90	< 0.001
Priority code in triage					
White	6,189	4.59	29,626	4.90	< 0.001
Green / Blue	94,731	70.33	411,049	68.05	< 0.001
Yellow / Orange	30,864	22.91	144,210	23.87	< 0.001
Red	2,917	2.17	19,278	3.19	< 0.001
Motivation for access					
Home, school or work accident	5,883	4.37	61,407	10.16	< 0.001
Sport/skiing accident	31,158	23.13	20,280	3.36	< 0.001
Road accident	5,599	4.16	19,399	3.21	< 0.001
Non traumatic	48,810	36.24	344,156	56.96	< 0.001
Trauma – accidental injury	41,940	31.14	152,401	25.23	< 0.001
Trauma – violence of others	981	0.73	4,444	0.74	
Other motivations	330	0.24	2,076	0.34	< 0.001
Hospital					
Arco – Garda Lake	14,980	11.12	55,058	9.11	< 0.001
Borgo – Valsugana	4,592	3.41	49,205	8.14	< 0.001
Cavalese – Dolomites	35,002	25.98	43,840	7.26	< 0.001
Cles – Val di Non / Val di Sole	18,750	13.92	72,175	11.95	< 0.001
Rovereto – Secondary Hub	15,268	11.33	109,336	18.10	< 0.001
Tione – Adamello Brenta	14,863	11.03	39,349	6.51	< 0.001
Trento – Hub	31,246	23.20	235,200	38.93	< 0.001
Year					
2018	19,631	14.57	85,869	14.21	< 0.01
2019	20,165	14.97	87,313	14.45	< 0.001
2020	14,280	10.60	72,677	12.03	< 0.001
2021	13,288	9.86	81,094	13.42	< 0.001
2022	20,860	15.49	90,733	15.02	< 0.001
2023	23,157	17.19	93,381	15.46	< 0.001
2024	23,320	17.31	93,096	15.41	< 0.001
Night time access					
Night (from 8pm to 8 am)	22,568	16.75	135,123	22.37	< 0.001
Day (from 8am to 8pm)	112,133	83.25	469,040	77.63	< 0.001
Days of the week					
Mon-Fri	94,921	70.47	442,327	73.21	< 0.001
Weekend	39,780	29.53	161,836	26.79	< 0.001

Resident and non-resident patients also had different patterns of access to the EDs. Non-residents were over-represented in hospitals located in touristic areas and under-represented elsewhere. Non-residents exhibited higher percentage of access during the weekend (29.5% vs. 26.8%, *p* < 0.01) and during the daytime (83.2% vs. 77.6%, *p* < 0.01). Finally, non-residents were under-represented in the pandemic years (2020: 10.6% vs. 12.0%, *p* < 0.01; 2021: 9.9% vs. 13.4%, *p* < 0.01) and overrepresented in all other years, with the largest differences in most recent years (2023: 17.2% vs. 15.5%, *p* < 0.01; 2024: 17.3% vs. 15.4%, *p* < 0.01).

### Differences in radiological examinations between resident and non-resident patients

Table 2 presents the differences between resident and non-resident patients with respect to radiological examinations. Over the 7 years of the study, the patients accessing the EDs generated an overall volume of 1,474,937 imaging examinations, of which 80% (1,180,240) were undertaken by residents and 20% (294,697) were undertaken by non-residents. On average, a resident patient accessing the ED underwent 1.95 radiological examination (among which 0.44 were CT), whereas a non-resident patient underwent 2.19 examination (among which 0.53 were CT). A lower percentage of non-resident patients undertook at least one CT scan (21.3% vs. 24.3%, *p* < 0.01) and at least one ultrasound (4.4% vs. 5.2%, *p* < 0.01). Non-resident patients undertook a higher percentage of skeletal x-rays (53.1% vs. 42.9%, *p* < 0.01) and CT (9.0% vs. 5.1%, *p* < 0.01), and abdomen CT (2.71% vs. 2.55%, *p* < 0.01), whereas they were under-represented in chest x-rays (17.6% vs. 25.9%, *p* < 0.01) and CT (0.27% vs. 0.35%, *p* < 0.01), head and neuro (9.4% vs. 12.4%, *p* < 0.01), abdomen x-rays (3.61% vs. 6.15%, *p* < 0.01), and ultrasound (2.6% vs. 3.3%, *p* < 0.01) examinations.

**Table 2:** Differences between resident and non-resident groups with respect to radiological examinations. CT, computed tomography. N, number

Radiological Examinations	Non Residents		Residents		*p*-value
	*N*	Percent	*N*	Percent	
Total exams	294,697		1,180,240		
Average exams per patient	2.1878		1.9535		
Average workload per patient	27.5853		24.4474		
Total CT	71,676		264,841		
Average CT per patient	0.5321		0.4384		
Patients with at least 1 CT	28,731	21.33	146,713	24.28	< 0.001
Patients with at least 1 Ultrasound	5,869	4.36	31,426	5.20	< 0.001
Type of imaging					
CT Angiography	522	0.18	2,957	0.25	< 0.001
CT Other	2,957	1.00	5,071	0.43	< 0.001
Specialty Ultrasound	668	0.23	4,019	0.34	< 0.001
Ultrasound	7,588	2.57	39,585	3.35	< 0.001
Abdomen X-Rays	10,626	3.61	72,581	6.15	< 0.001
Chest X-Rays	51,821	17.58	305,716	25.90	< 0.001
Skeletal X-Rays	156,545	53.12	506,560	42.92	< 0.001
Abdomen CT	7,999	2.71	30,056	2.55	< 0.001
Chest CT	795	0.27	4,112	0.35	< 0.001
Head/Neuro CT	27,813	9.44	146,250	12.39	< 0.001
Skeletal CT	26,565	9.01	60,085	5.09	< 0.001
Other	798	0.27	3,248	0.28	

### Regression models to assess the difference in the number of radiological examinations between resident and non-resident patients

.

**Fig. 1:**
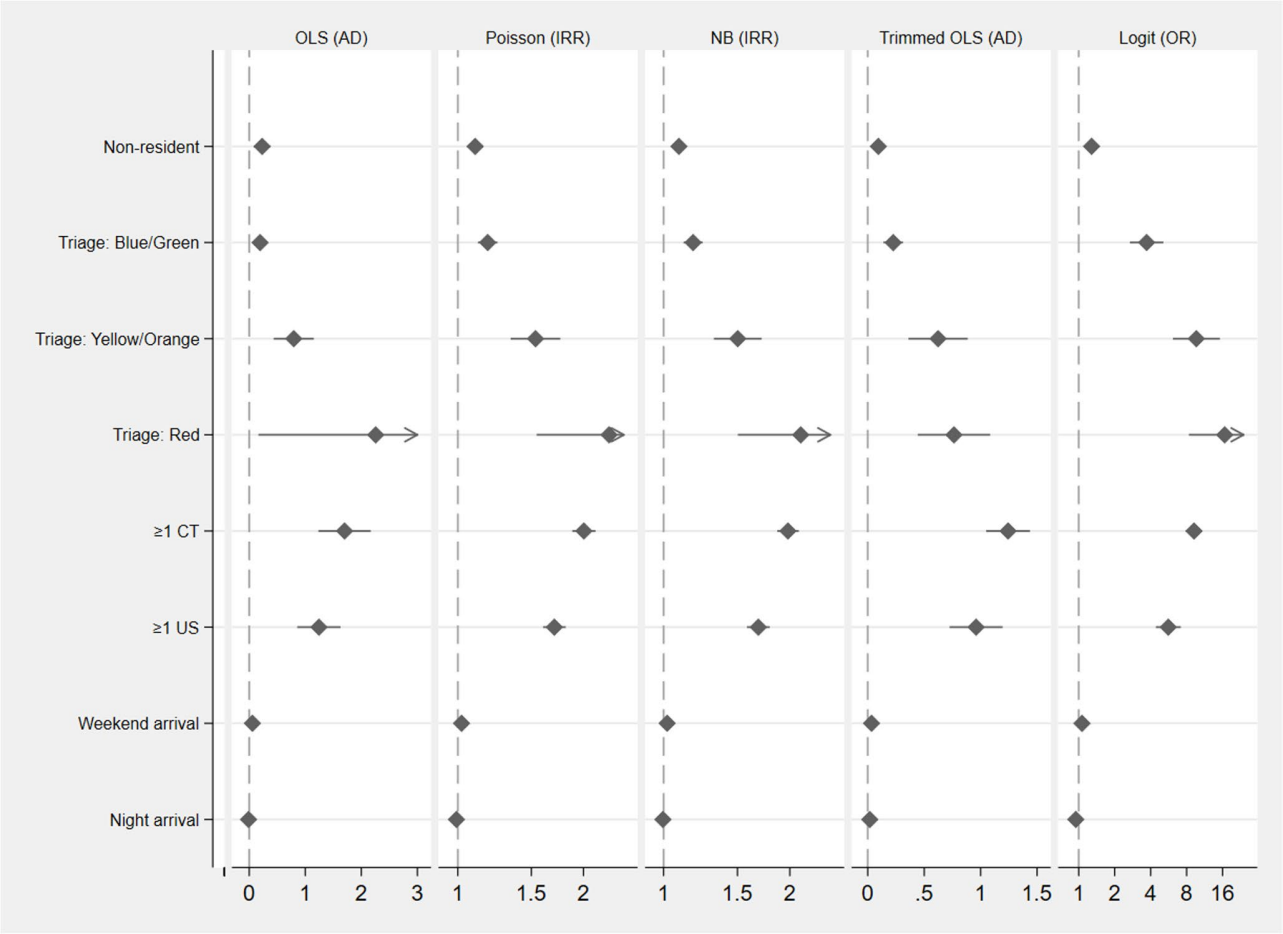
Adjusted associations of patient and ED access factors with the number ofradiological exams, with 95% confidence intervals; estimates control for hospital,year, age, and motivation fixed effects; standard errors are clustered at hospital-yearlevel. Adjusted differences in number of exams are reported for OLS panels;incidence rate ratios (IRR) in log scale are reported for Poisson and NB panels; oddsratios (OR) in log scale are reported for Logit panel. Confidence intervals for RedTriage variable are truncated at axis limit: arrow indicates continuation. CT,computed tomography; ED, emergency department; NB, negative binomial; OLS,ordinary least squares; US, ultrasound

Figure 1 reports the main results from the regression models of the number of radiological examinations. More specifically, it reports adjusted differences for the OLS and Trimmed OLS models, incidence rate ratios (IRR) for the Poisson and NB models, and odds ratios (OR) for the Logit model. Qualitative results were consistent across all five models: (1) patients with higher priority levels underwent more imaging; (2) patients taking at least 1 CT or 1 ultrasound exam underwent more imaging; (3) patients accessing ED during weekends underwent more imaging. The following results were not explicitly reported in Fig. 1, but emerged consistently from the models: (1) the number of exams undertaken by each patient was larger over the pandemic years (2020–2022); (2) the number of exams undertaken by each patient was larger if access occurred at the hub hospital in Trento; (3) the number of exams undertaken increased with the age of the patient; (4) the number of exams undertaken was higher if the motivation for ED access was a road accident and lower if it was a non-traumatic event (see Table S1 in supplementary materials).

The results from the OLS model showed that after controlling for all demographic, clinical, and access variables that differ across resident and non-resident patients, a patient from the latter group still underwent 0.23 more examinations than a resident patient with the same characteristics. This value is slightly lower when using the Poisson model, as the estimated coefficient implies that non-residents underwent about 10% more examinations than resident patients (0.19 more imaging).

Overdispersion was detected in the data through multiple tests:1) the Variance-to-Mean ratio was equal to 3.40; 2) the Goodness-of-fit tests indicated significant overdispersion in the Poisson model (Pearson χ² = 955,349.9, df = 738,830, *p* < 0.01; Deviance = 635,868.1, df = 738,830, *p* = 1); 3) the Cameron-Trivedi test was highly significant (α̂ = 0.531, *p* < 0.01), confirming substantial overdispersion in the Poisson model. When using the NB model, the estimated coefficient implied that non-residents underwent about 8.8% more examinations than resident patients (0.17 more imaging). When considering the restricted sample of the Trimmed OLS model, the difference in the number of examinations between residents and non-residents further decreased to 0.09. Finally, from the coefficient estimated in the Logit model emerged that non-residents had 31.9% higher odds of being in the group of patients who underwent a disproportionately larger number of radiological examinations (top 5%).

### Interaction between resident status and triage priority code

**Fig. 2:**
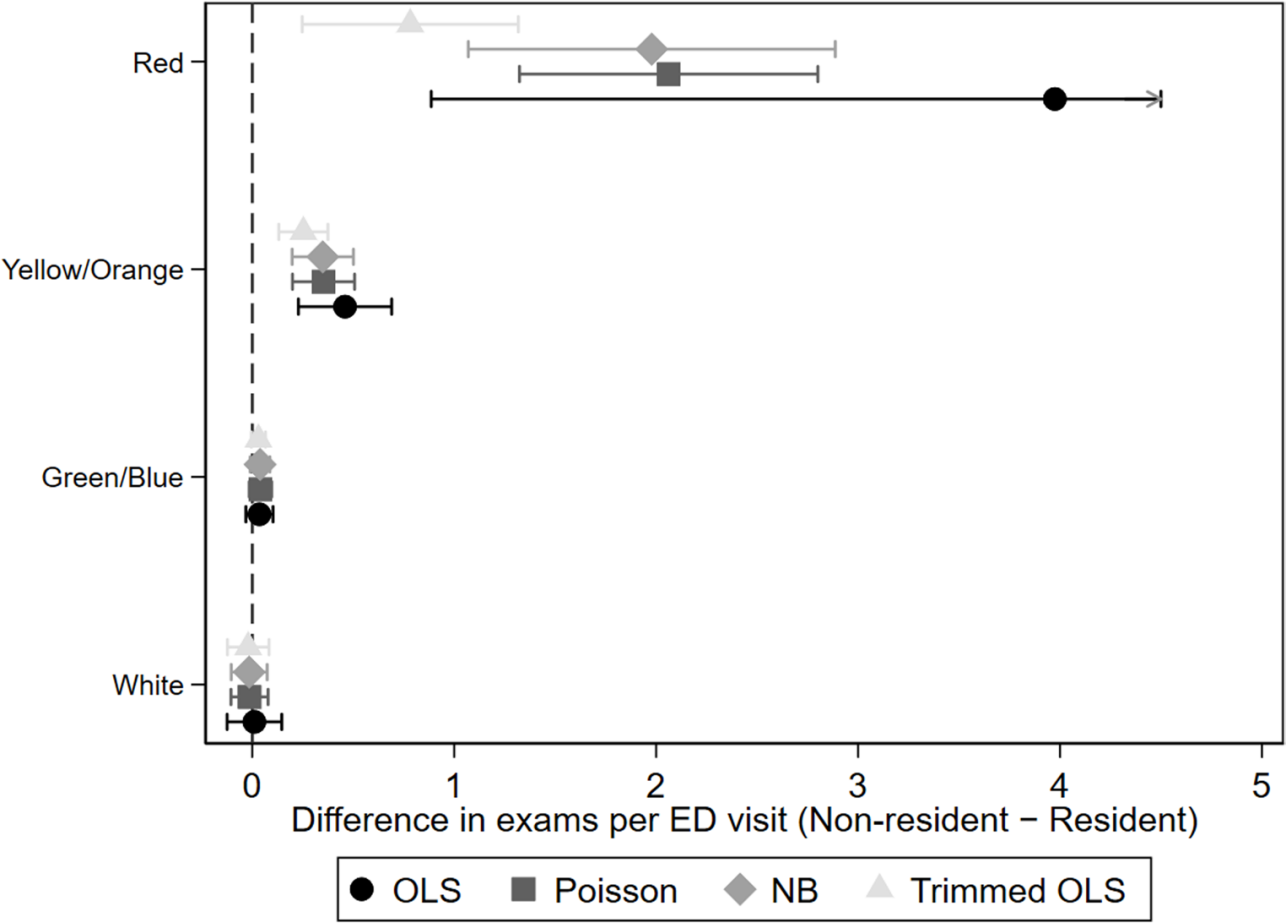
Differences in the number of exams per ED access between non-residents andresidents, by triage priority code, with 95% confidence intervals. Average marginaleffects estimated from the following four models with interaction between residencestatus and triage priority code: OLS, Poisson, NB, Trimmed OLS. Estimates controlfor weekend arrival, nighttime arrival, undertaking at least 1 CT, undertaking at least1 US, and for hospital, year, age, and motivation fixed effects; standard errors areclustered at hospital-year level. Confidence intervals for Red Triage variable in theOLS model is truncated at axis limit: arrow indicates continuation. CT, computedtomography; ED, emergency department; NB, negative binomial; OLS, ordinary leastsquares; US, ultrasound

**Fig. 3:**
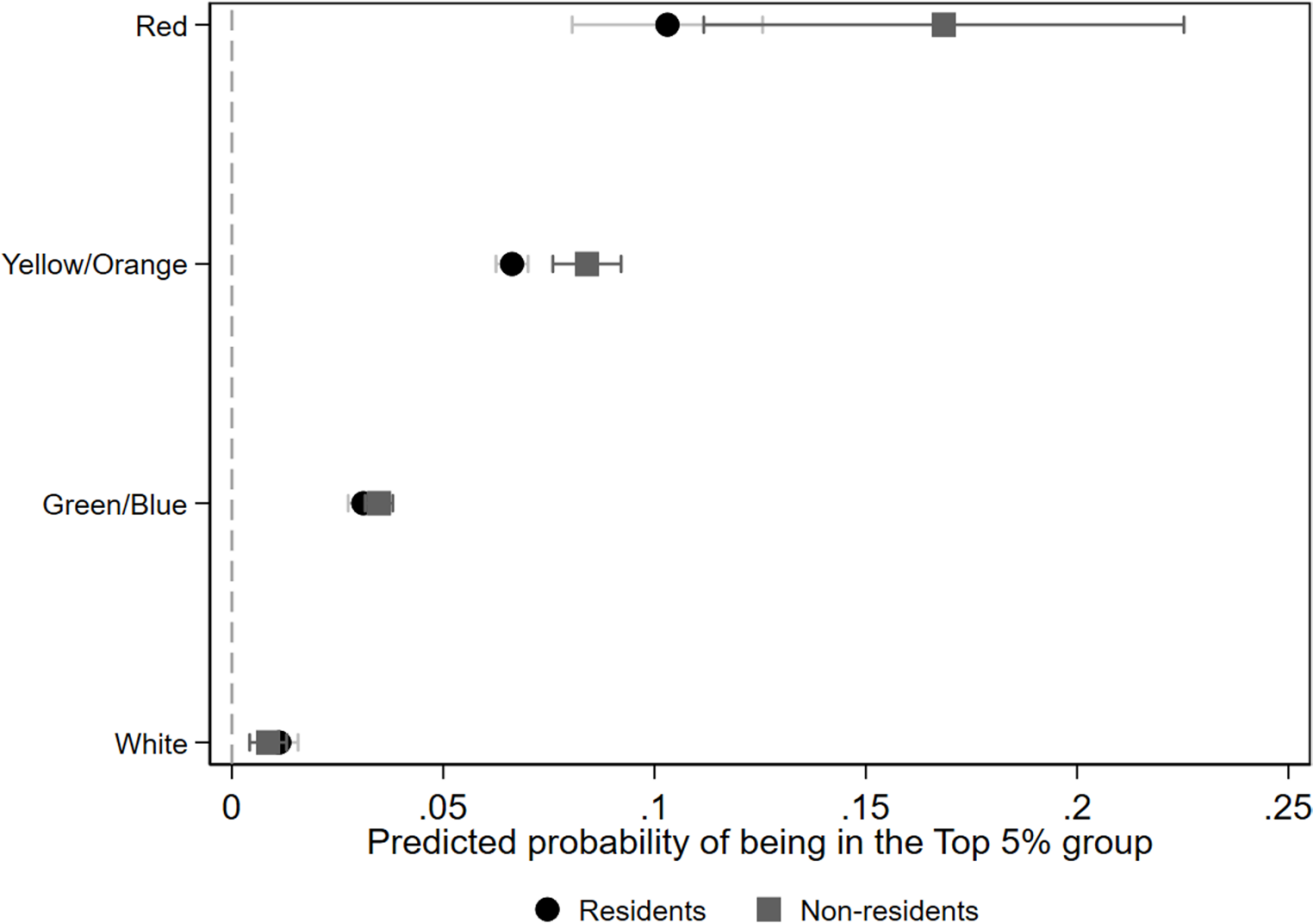
Predicted probabilities of being in the top 5% group by number of exams, byresidence status and triage priority code, with 95% confidence intervals. Logit modelwith interaction between residence status and triage priority code. Estimates controlfor weekend arrival, nighttime arrival, undertaking at least 1 CT, undertaking at least1 US, and for hospital, year, age, and motivation fixed effects; standard errors areclustered at hospital-year level. CT, computed tomography; US, ultrasound

Figure 2 reports the main results from the OLS, Poisson, NB, and Trimmed OLS regression models with an interaction term between the resident status dummy and the triage priority code categorical variables (see Table S2 in supplementary materials). Table 3 summarizes the estimated percentage changes in the number of examinations due to the residence status of the patients emerging from the different models. Consistently across all models, the results showed that the difference in the number of examinations undertaken by residents and non-residents was driven by patients with yellow/orange code and red code, whereas the difference for patients with a white or green/blue code was minimal in magnitude and statistically not significant. The quantitative effect depends on the estimation method: for the yellow/orange code it ranges from 0.46 more exams (27%) for non-residents using OLS to 0.35 (17%) using the NB model; for the red code, it ranges from 3.97 more exams (145%) for non-residents using OLS to 1.98 (66%) using the NB model. The results from the Logit model also showed that the difference in the probability of undertaking a disproportionately larger number of radiological examinations (top 5%) between non-residents and residents depended on the triage priority codes. Figure 3 reports the predicted probabilities of being in the top 5% group: there was no significant difference between residents and non-residents who received a white code; patients with a blue/green code had a 3.1% probability if resident and a 3.5% probability if non-resident; patients with a yellow/orange code had a 6.6% probability if resident and a 8.4% probability if non-resident; patients with a red code had a 10.3% probability if resident and a 16.8% probability if non-residents.

**Table 3:** Estimated percentage difference in the number of imaging examinations due to the residence status of the patients emerging from different models. OLS, ordinary least squares. NB, negative binomial

Triage Code	OLS	Poisson	NB	Trimmed OLS
White	+ 1%	-1%	-1%	-4%
Green / Blue	+ 3%	+ 3%	+ 3%	-2%
Yellow / Orange	+ 27%	+ 17%	+ 17%	+ 22%
Red	+ 145%	+ 66%	+ 66%	+ 60%

### Heterogeneity in examination complexity

Results about the heterogeneity in examination complexity are reported in the supplementary materials. Tables S3 and S4 report the full results from the regression models of the number of CT examinations on resident status (Table S3), and with an interaction term between the resident status dummy and the triage priority code categorical variables (Table S4). Figure S1 summarizes the main results: non-residents undertake more CT examinations compared to resident patients, with an effect that ranges between 37.1% (0.16 more CTs per patient) when OLS is used for estimation to 24.8% (0.11 more CTs per patient) obtained with the NB model, and which is generally stronger compared to the case with all examinations. For high-acuity cases accessing ED with a red triage code, the difference between residents and non-residents was higher, and ranged between 162% (3.5 more CTs per patient) with OLS estimation and 117% (2.52 more CTs per patient) with Poisson estimation. Table S5 and S6 report instead the full results of regressions considering the time-weighted imaging burden. The results for the resident status variable are summarized in Figure S2: they show that after controlling for triage code, age, motivation for access, and other relevant factors, non-resident patient still generate a higher workload on radiologists that can be quantified in about 4 min per patient (15.5% increase) using OLS, or slightly smaller values using alternative estimation models (Poisson: 12.6%; NB: 7.6%; Trimmed OLS: 6.1%).

## Discussion

This study analyzed a very large dataset of ED patients from a highly touristic province in Northern Italy, evaluating the differences in the use of radiological examinations between resident and non-resident patients. We document significant differences between these two populations, and in particular we report four major findings.

First, resident and non-resident patients differ significantly in their demographic, clinical, and access characteristics. Non-residents are more often male and younger, diverging from trends in recent literature suggesting an aging tourist population [8, 19]. However, this aligns with the predominance of sport-related tourism in the Trento province and similar findings in areas focused on sport tourism [4, 20, 21]. The clinical motivations for ED visits, as well as temporal and spatial patterns that we found, also align with tourism behavior.

Second, this is the first study providing evidence about the differential use of ED radiological services by residence status. Non-residents account for approximately 20% of all ED radiological exams during the study period in the Province of Trento. Their exam profile is skewed toward skeletal imaging, consistent with the traumatic nature of their ED access. This finding highlights the pressure that tourism can exert on specific hospital services, particularly diagnostic imaging.

Third, even after controlling for demographic, clinical, and access-related factors, non-residents undergo more general radiological and CT exams, and determine a higher imaging workload per ED access than residents. Across models, the excess ranges from 0.09 to 0.23 examination per patient, from 0.04 to 0.16 CTs per patient, and from 1.26 to 3.46 min per patient, corresponding to an annual surplus of 3,233 to 4,426 examinations, of 760 to 3,079 CTs, and of 459 to 1212 h determined by residence status alone. This surplus is in addition to the higher baseline demand for radiological services due to non-residents’ distinct clinical and demographic profiles. These results suggest a more intensive diagnostic approach for non-residents, independent of their clinical condition.

Fourth, the difference in the use of radiological services is modulated by triage priority. Among patients with low-priority triage codes (white or green/blue), the difference between residents and non-residents is negligible. However, for high-acuity cases (yellow/orange or red priority codes), the difference becomes substantial: non-residents with a yellow/orange code receive, on average, about half an exam more than residents, while non-residents with a red code receive nearly four more exams. In the case of red codes, almost all of the increase in the number of exams is generated by CTs. These patterns are consistent across regression models.

Together, these findings indicate that non-resident patients – particularly those in serious clinical condition – receive more extensive radiological assessment. A potential explanation is the limited clinical history available for non-residents, which may prompt more comprehensive diagnostic imaging. Language barriers and communication challenges may also play a role in extending diagnostic procedures [3, 6]. These factors could lead to a cautious approach by clinicians in order to minimize diagnostic uncertainty.

This study benefits from a large and robust dataset covering all EDs in a highly touristic region over a seven-year period. While the dataset has many strengths, it also has some weaknesses. First, we do not have information about the actual status of tourist patients, and we need to rely on residence information as a proxy for the tourist status. Although this approach captures non-resident patients accessing emergency services, it may introduce some degree of misclassification, as not all non-residents are necessarily tourists and some tourists may temporarily reside within the region. Second, we also lack individual-level information on comorbidities, clinical conditions and outcomes, diagnostic yield, and imaging appropriateness: therefore, we cannot determine whether the higher imaging intensity observed among non-residents reflects appropriate diagnostic caution, differences in clinical complexity, or potential overutilization. Third, our analysis does not account for provider-level factors or institutional protocols that may influence radiological decision-making. Fourth, while statistically robust, the absolute differences in imaging volume per patient are relatively modest. However, these differences should be interpreted in the broader context of cumulative system-level impact, where even small increases per patient may translate into substantial additional workload for radiology services considering the seasonal nature of the tourism phenomenon and stronger impact for high-priority cases. Finally, an additional limitation concerns the external validity of our findings. The Autonomous Province of Trento represents a highly specific tourism environment characterized by strong seasonal influxes associated with alpine sport activities, particularly skiing and mountain sports. As a result, the clinical profile of non-resident patients is heavily influenced by trauma-related presentations. This context likely contributes to the high proportion of skeletal imaging and CT examinations observed among non-residents. Imaging demand patterns in this setting may therefore differ from those observed in other tourism models, such as coastal destinations dominated by summer leisure travel [6], urban cultural centers, or regions where visitors more commonly present with non-traumatic medical conditions [8]. In these contexts, imaging utilization may be driven more by cardiopulmonary, gastrointestinal, or infectious presentations rather than trauma. Although we control for observable factors such as age or motivation for ED access, caution is warranted when extrapolating these results to other tourism environments. Future studies conducted in different geographic and tourism contexts would be valuable to assess whether the relationship between residence status and imaging intensity observed here generalizes to other emergency care systems.

Still, as tourism continues to grow in regions like Trentino as well as other alpine areas [22], understanding and managing its impact on healthcare services is essential. Indeed, our results can provide useful implications for health and tourism policy. From an operational perspective, these findings have implications for staffing and resource planning in emergency radiology departments serving high-tourism regions. Seasonal increases in trauma-related emergency visits among non-residents may generate disproportionate demand for specific imaging modalities, particularly skeletal radiography and CT. Radiology departments located near major tourist hubs may therefore benefit from adaptive staffing models that account for seasonal population fluctuations, including flexible scheduling of personnel during peak tourism periods. In addition, anticipating modality-specific surges may support improved allocation of scanner time, prioritization protocols, and workflow management within emergency radiology units. Integrating tourism flow data into hospital planning models could further enhance the ability of healthcare systems to anticipate demand and maintain efficient diagnostic pathways during high-volume periods. Further research is needed to explore whether increased imaging demand among non-residents improves clinical outcomes or simply reflects greater diagnostic uncertainty, and in the latter case, what are the sources of this uncertainty and which strategies can be implemented to reduce unnecessary imaging examinations [23–25]. 

## Supplementary Information

Below is the link to the electronic supplementary material.

Supplementary Material 1

## Data Availability

The authors do not have permission to share data.
