# Supplementary material for: Emergency radiology demand in a touristic region: residence status as a driver of imaging use
Source: Emerg Radiol. 2026 Apr 17;33(4):675–85. doi: 10.1007/s10140-026-02464-4 (PMC13433424; doi:10.1007/s10140-026-02464-4)
Supplement: Supplementary file 1 [file 10140_2026_2464_MOESM1_ESM.docx]

**Supplementary material**

|  | Model 1 | Model 2 | Model 3 | Model 4 | Model 5 |
| --- | --- | --- | --- | --- | --- |
| Estimation | OLS | Poisson | NB | Trimmed OLS | Logit |
| D.V. | # Exams | # Exams | # Exams | # Exams | Top 5%  (# Exams) |
| Resident | -0.2305^**^  (0.0744) | -0.0953^***^  (0.0225) | -0.0844^***^  (0.0191) | -0.0937^***^  (0.0246) | -0.2767^***^  (0.0576) |
| Priority Code  (*Ref. White*) |  |  |  |  |  |
| Green/Blue | 0.1932^**^  (0.0518) | 0.1647^***^  (0.0276) | 0.1621^***^  (0.0267) | 0.2254^***^  (0.043) | 1.2266^***^  (0.1875) |
| Yellow/Orange | 0.7949^***^  (0.178) | 0.4282^***^  (0.07) | 0.4071^***^  (0.067) | 0.6231^***^  (0.1306) | 2.222^***^  (0.2552) |
| Red | 2.256^*^  (1.0399) | 0.8347^***^  (0.2041) | 0.7529^***^  (0.1761) | 0.7635^***^  (0.1596) | 2.918^***^  (0.3914) |
| At least 1 CT | 1.7009^***^  (0.2324) | 0.6953^***^  (0.0327) | 0.6829^***^  (0.0302) | 1.243^***^  (0.0965) | 2.3954^***^  (0.0851) |
| At least 1 US | 1.2435^***^  (0.1922) | 0.5325^***^  (0.0318) | 0.5202^***^  (0.032) | 0.9598^***^  (0.117) | 1.6442^***^  (0.1001) |
| Male | -0.0043  (0.0307) | -0.0098  (0.0125) | -0.0149  (0.0111) | -0.0496^*^  (0.0198) | -0.0359  (0.0445) |
| Weekend | 0.0565^*^  (0.0233) | 0.0208^*^  (0.0081) | 0.0197^*^  (0.0077) | 0.0327^*^  (0.0146) | 0.0397  (0.0372) |
| Night | -0.0121  (0.0263) | -0.0077  (0.0122) | -0.0037  (0.0118) | 0.0173  (0.0197) | -0.0827^*^  (0.0396) |
| Constant | 1.1711  (0.7615) | 0.0793  (0.2402) | 0.0882  (0.2265) | 0.6273^**^  (0.1994) | -7.1058^***^  (0.4559) |
| Year FE | Yes | Yes | Yes | Yes | Yes |
| Motivation FE | Yes | Yes | Yes | Yes | Yes |
| Age Category FE | Yes | Yes | Yes | Yes | Yes |
| Hospital FE | Yes | Yes | Yes | Yes | Yes |
| R^2^ | 0.198 |  |  | 0.2545 |  |
| Pseudo R^2^ |  | 0.1498 | 0.1066 |  | 0.3086 |
| Obs | 738,864 | 738,864 | 738,864 | 733,510 | 738,864 |

**Table S1.** Regression models to assess the difference in the number of radiological examinations between resident and non-resident groups. Coefficients are reported for all models; standard errors clustered at hospital-year level in parentheses. CT, computed tomography; NB, negative binomial; OLS, ordinary least squares; US, ultrasound.

|  | Model 1 | Model 2 | Model 3 | Model 4 | Model 5 |
| --- | --- | --- | --- | --- | --- |
| Estimation | OLS | Poisson | NB | Trimmed OLS | Logit |
| D.V. | # Exams | # Exams | # Exams | # Exams | Top 5%  (# Exams) |
| Resident | -0.0105  (0.0672) | 0.0091 (0.0309) | 0.0101  (0.0297) | 0.0203  (0.0512) | 0.2909  (0.1911) |
| Priority Code (*Ref. White*) |  |  |  |  |  |
| Green / Blue | 0.2188^*^  (0.0826) | 0.1927^***^  (0.0407) | 0.1897^***^  (0.0391) | 0.2685^***^  (0.0653) | 1.5864^***^  (0.2641) |
| Yellow / Orange | 1.1694^***^  (0.2222) | 0.5545^***^  (0.0743) | 0.5346^***^  (0.0717) | 0.849^***^  (0.1472) | 2.7329^***^  (0.3035) |
| Red | 5.6984^*^  (2.2469) | 1.241^***^  (0.1729) | 1.178^***^  (0.1821) | 1.4684^***^  (0.3664) | 3.7824^***^  (0.5026) |
| Green / Blue * Resident | -0.0249  (0.055) | -0.0313  (0.0264) | -0.0311  (0.0253) | -0.0506  (0.0431) | -0.4264^*^  (0.2017) |
| Yellow / Orange * Resident | -0.4487^**^  (0.1287) | -0.1528^***^  (0.0393) | -0.1542^***^  (0.0391) | -0.2731^**^  (0.0854) | -0.6161^**^  (0.2064) |
| Red *  Resident | -3.9643^*^  (1.521) | -0.5099^***^  (0.0781) | -0.5204^***^  (0.0733) | -0.8026^**^  (0.2663) | -1.0483^***^  (0.2795) |
| At least 1 CT | 1.68^***^  (0.2239) | 0.6903^***^  (0.0321) | 0.6784^***^  (0.0296) | 1.2387^***^  (0.0955) | 2.3895^***^  (0.084) |
| At least 1 US | 1.2348^***^  (0.1885) | 0.5299^***^  (0.0316) | 0.518^***^  (0.0319) | 0.9575^***^  (0.1165) | 1.6415^***^  (0.1) |
| Male | -0.0117 (0.0287) | -0.0125 (0.0121) | -0.0173  (0.0108) | -0.0516^*^  (0.0196) | -0.0389  (0.0443) |
| Weekend | 0.0528^*^  (0.0216) | 0.0194^*^  (0.0077) | 0.0183^*^  (0.0075) | 0.0316^*^  (0.0146) | 0.039  (0.0371) |
| Night | -0.0104 (0.0259) | -0.0066  (0.0121) | -0.0026 (0.0117) | 0.018  (0.0197) | -0.0818^*^  (0.0399) |
| Constant | 1.0044  (0.7755) | 0.0032  (0.2442) | 0.0209  (0.2301) | 0.5326^*^  (0.1998) | -7.5647^***^  (0.4935) |
| Year FE | Yes | Yes | Yes | Yes | Yes |
| Motivation FE | Yes | Yes | Yes | Yes | Yes |
| Age Category FE | Yes | Yes | Yes | Yes | Yes |
| Hospital FE | Yes | Yes | Yes | Yes | Yes |
| R^2^ | 0.206 |  |  | 0.2557 |  |
| Pseudo R^2^ |  | 0.1511 | 0.1076 |  | 0.3091 |
| Obs | 738,864 | 738,864 | 738,864 | 733,510 | 738,864 |

**Table S2.** Regression models to assess the difference in the number of radiological examinations between resident and non-resident groups, with the addition of an interaction term between the resident status dummy and the triage priority code categorical variables. Coefficients are reported for all models; standard errors clustered at hospital-year level in parentheses. CT, computed tomography; OLS, ordinary least squares; US, ultrasound.

|  | Model 1 | Model 2 | Model 3 | Model 4 | Model 5 |
| --- | --- | --- | --- | --- | --- |
| Estimation | OLS | Poisson | NB | Trimmed OLS | Logit |
| D.V. | # CT | # CT | # CT | # CT | Top 5%  (# CT) |
| Resident | -0.1627^*^  (0.0642) | -0.2737^***^  (0.0627) | -0.2219^***^  (0.06) | -0.0334^**^  (0.0094) | -0.3905^***^  (0.077) |
| Priority Code  (*Ref. White*) |  |  |  |  |  |
| Green/Blue | 0.0693^*^  (0.0261) | 0.625^***^  (0.1124) | 0.627^***^  (0.1007) | 0.0914^***^  (0.0079) | 0.8282^**^  (0.2608) |
| Yellow/Orange | 0.5992^***^  (0.1067) | 1.6958^***^  (0.2557) | 1.6018^***^  (0.2322) | 0.4022^***^  (0.0378) | 2.531^***^  (0.4051) |
| Red | 2.363^*^  (0.8934) | 2.7324^***^  (0.4262) | 2.547^***^  (0.4018) | 0.8377^***^  (0.1007) | 3.8741^***^  (0.595) |
| At least 1 US | -0.1521  (0.0714) | -0.1693  (0.1932) | -0.1907  (0.214) | -0.0648^***^  (0.0603) | 0.751  (0.2285) |
| Male | 0.0501  (0.0296) | 0.0934  (0.0501) | 0.0284  (0.03) | -0.0072  (0.0075) | 0.2198^***^  (0.0596) |
| Weekend | 0.0257  (0.0191) | 0.0375  (0.0249) | 0.0259  (0.0289) | 0.0002  (0.008) | 0.0205  (0.0474) |
| Night | -0.0467^*^  (0.0187) | -0.0846  (0.0436) | -0.0601  (0.0514) | -0.0167  (0.0189) | -0.1423^*^  (0.0704) |
| Constant | 0.3268  (0.5041) | -3.2655^***^  (0.4825) | 3.4021^***^  (0.4743) | 0.0604  (0.0684) | -7.0804^***^  (0.7292) |
| Year FE | Yes | Yes | Yes | Yes | Yes |
| Motivation FE | Yes | Yes | Yes | Yes | Yes |
| Age Category FE | Yes | Yes | Yes | Yes | Yes |
| Hospital FE | Yes | Yes | Yes | Yes | Yes |
| R^2^ | 0.1169 |  |  | 0.0938 |  |
| Pseudo R^2^ |  | 0.1996 | 0.0915 |  | 0.1941 |
| Obs | 738,864 | 738,864 | 738,864 | 732,763 | 738,864 |

**Table S3.** Regression models to assess the difference in the number of CT examinations between resident and non-resident groups. Coefficients are reported for all models; standard errors clustered at hospital-year level in parentheses. CT, computed tomography; NB, negative binomial; OLS, ordinary least squares; US, ultrasound.

|  | Model 1 | Model 2 | Model 3 | Model 4 | Model 5 |
| --- | --- | --- | --- | --- | --- |
| Estimation | OLS | Poisson | NB | Trimmed OLS | Logit |
| D.V. | # CT | # CT | # CT | # CT | Top 5%  (# CT) |
| Resident | -0.0204  (0.0234) | 0.0679 (0.1139) | 0.0743  (0.1034) | 0.0034  (0.0107) | 0.3074  (0.2832) |
| Priority Code (*Ref. White*) |  |  |  |  |  |
| Green / Blue | 0.0537  (0.0279) | 0.6101^***^  (0.124) | 0.6615^***^  (0.1165) | 0.0925^***^  (0.0116) | 1.1295^**^  (0.4203 |
| Yellow / Orange | 0.8497^***^  (0.1575) | 2.0583^***^  (0.2092) | 2.058^***^  (0.2141) | 0.5028^***^  (0.0372) | 3.1584^***^  (0.4514) |
| Red | 5.3985^**^  (1.9114) | 3.3435^***^  (0.3145) | 3.4235^***^  (0.4123) | 1.2512^***^  (0.1806) | 4.9388^***^  (0.6524) |
| Green / Blue * Resident | 0.0227  (0.0156) | 0.0243  (0.0965) | -0.0398  (0.0841) | -0.0007  (0.0086) | -0.345  (0.3029) |
| Yellow / Orange * Resident | -0.3038^**^  (0.0965) | -0.444^***^  (0.1219) | -0.5561^***^  (0.1144) | -0.1221^***^  (0.031) | -0.7609^**^  (0.2591) |
| Red *  Resident | -3.5012^**^  (1.2899) | -0.7728^**^  (0.2496) | -1.0607^***^  (0.1794) | -0.4657^***^  (0.0988) | -1.2891^***^  (0.2826) |
| At least 1 US | -0.0212  (0.0684) | -0.1744  (0.1934) | -0.195  (0.2148) | 0.0659  (0.0602) | 0.0711  (0.2276) |
| Male | -0.044 (0.0273) | 0.0857 (0.0503) | -0.0226  (0.0301) | -0.0083  (0.0075) | 0.2139^***^  (0.06) |
| Weekend | 0.0226  (0.0174) | 0.0329  (0.0245) | 0.0214  (0.0285) | -0.0003  (0.008) | 0.0185  (0.0479) |
| Night | -0.0453^*^ (0.0189) | -0.0812  (0.0445) | -0.0553 (0.0517) | -0.0163  (0.019) | -0.1412^*^  (0.0709) |
| Constant | 0.2233  (0.5191) | -3.5213^***^  (0.4552) | 3.6207  (0.4597) | 0.0915  (0.0644) | -7.6495^***^  (0.7873) |
| Year FE | Yes | Yes | Yes | Yes | Yes |
| Motivation FE | Yes | Yes | Yes | Yes | Yes |
| Age Category FE | Yes | Yes | Yes | Yes | Yes |
| Hospital FE | Yes | Yes | Yes | Yes | Yes |
| R^2^ | 0.1335 |  |  | 0.0952 |  |
| Pseudo R^2^ |  | 0.2025 | 0.0932 |  | 0.1955 |
| Obs | 738,864 | 738,864 | 738,864 | 732,763 | 738,864 |

**Table S4.** Regression models to assess the difference in the number of CT examinations between resident and non-resident groups, with the addition of an interaction term between the resident status dummy and the triage priority code categorical variables. Coefficients are reported for all models; standard errors clustered at hospital-year level in parentheses. CT, computed tomography; OLS, ordinary least squares; US, ultrasound.

|  | Model 1 | Model 2 | Model 3 | Model 4 |
| --- | --- | --- | --- | --- |
| Estimation | OLS | Poisson | NB | Trimmed OLS |
| D.V. | Time-Weighted Imaging Burden | Time-Weighted Imaging Burden | Time-Weighted Imaging Burden | Time-Weighted Imaging Burden |
| Resident | -3.7792^**^  (1.335) | -0.1187^***^  (0.0288) | -0.0734^***^  (0.0173) | -1.43^**^  (0.3927) |
| Priority Code  (*Ref. White*) |  |  |  |  |
| Green/Blue | 1.3644  (0.7479) | 0.1317^***^  (0.03) | 0.1203^***^  (0.0257) | 1.9774^***^  (0.5094) |
| Yellow/Orange | 9.7898^***^  (2.2719) | 0.4094^***^  (0.0758) | 0.3201^***^  (0.069) | 7.3048^***^  (1.5312) |
| Red | 41.1868^*^  (18.7929) | 0.9759^***^  (0.2165) | 0.7059^***^  (0.1793) | 14.1274^***^  (3.1819) |
| At least 1 CT | 32. 0517^***^  (3.4442) | 1.0158^***^  (0.0319) | 1.0081^***^  (0.0289) | 24.9838^***^  (1.2464) |
| At least 1 US | 33.8246^***^  (2.3831) | 0.9963^***^  (0.0419) | 1.0708^***^  (0.0128) | 29.633^***^  (1.326) |
| Male | 0.7566  (0.5295) | 0.0129  (0.0148) | -0.0167  (0.0097) | -0.135  (0.2504) |
| Weekend | 0.7442  (0.4008) | 0.0174  (0.01) | 0.0135  (0.0078) | 0.3021  (0.2142) |
| Night | -0.5008  (0.363) | -0.0196  (0.0121) | -0.0042  (0.01) | 0.0382  (0.2184) |
| Constant | 15.6472  (12.3787) | 2.5367^***^  (0.262) | 2.537^***^  (0.2136) | 6.3123^*^  (2.4803) |
| Year FE | Yes | Yes | Yes | Yes |
| Motivation FE | Yes | Yes | Yes | Yes |
| Age Category FE | Yes | Yes | Yes | Yes |
| Hospital FE | Yes | Yes | Yes | Yes |
| R^2^ | 0.2393 |  |  | 0.3699 |
| Pseudo R^2^ |  | 0.4369 | 0.1031 |  |
| Obs | 738,864 | 738,864 | 738,864 | 733,510 |

**Table S5.** Regression models to assess the difference in the time-weighted imaging burden of radiological examinations between resident and non-resident groups. Coefficients are reported for all models; standard errors clustered at hospital-year level in parentheses. CT, computed tomography; NB, negative binomial; OLS, ordinary least squares; US, ultrasound.

|  | Model 1 | Model 2 | Model 3 | Model 4 |
| --- | --- | --- | --- | --- |
| Estimation | OLS | Poisson | NB | Trimmed OLS |
| D.V. | Time-Weighted Imaging Burden | Time-Weighted Imaging Burden | Time-Weighted Imaging Burden | Time-Weighted Imaging Burden |
| Resident | -0.1235  (0.8403) | 0.024 (0.0313) | 0.0172  (0.025) | 0.3605  (0.5737) |
| Priority Code (*Ref. White*) |  |  |  |  |
| Green / Blue | 1.7584  (1.0981) | 0.1734^***^  (0.0417) | 0.1537^***^  (0.0339) | 2.6504^**^  (0.7499) |
| Yellow / Orange | 15.3504^***^  (3.3345) | 0.5639^***^  (0.086) | 0.4659^***^  (0.0783) | 10.5151^***^  (1.9323) |
| Red | 105.913^*^  (40.9192) | 1.4078^***^  (0.1733) | 1.2459^***^  (0.2205) | 29.2712^***^  (7.1302) |
| Green / Blue * Resident | -0.3687  (0.6853) | -0.0472  (0.026) | -0.0382  (0.021) | -0.7896  (0.4871) |
| Yellow / Orange * Resident | -6.635^**^  (2.0725) | -0.1857^***^  (0.0413) | -0.1748^***^  (0.0397) | -3.8756^**^  (1.1464) |
| Red *  Resident | -74.5092^*^  (27.9361) | -0.5461^***^  (0.097) | -0.6453^***^  (0.1013) | -17.1842^**^  (5.246) |
| At least 1 CT | 31.6739^***^  (3.2747) | 1.0103^***^  (0.0313) | 1.0035^***^  (0.0282) | 24.9059^***^  (1.2227) |
| At least 1 US | 33.6732^***^  (2.3134) | 0.9933^***^  (0.0413) | 1.0686^***^  (0.0125) | 29.5962^***^  (1.3153) |
| Male | 0.6287 (0.4843) | 0.0099 (0.0145) | -0.0185  (0.0096) | -0.1682^*^  (0.2462) |
| Weekend | 0.6818  (0.3646) | 0.0156  (0.0094) | 0.0121  (0.0077) | 0.286  (0.2126) |
| Night | -0.4752 (0.3483) | -0.0182  (0.0121) | -0.003 (0.0099) | 0.0465  (0.2192) |
| Constant | 12.9108  (12.6942) | 2.432  (0.2674) | 2.4771  (0.2172) | 4.8258  (2.5234) |
| Year FE | Yes | Yes | Yes | Yes |
| Motivation FE | Yes | Yes | Yes | Yes |
| Age Category FE | Yes | Yes | Yes | Yes |
| Hospital FE | Yes | Yes | Yes | Yes |
| R^2^ | 0.2505 |  |  | 0.3718 |
| Pseudo R^2^ |  | 0.4399 | 0.1038 |  |
| Obs | 738,864 | 738,864 | 738,864 | 733,510 |

**Table S6.** Regression models to assess the difference in the time-weighted imaging burden of radiological examinations between resident and non-resident groups, with the addition of an interaction term between the resident status dummy and the triage priority code categorical variables. Coefficients are reported for all models; standard errors clustered at hospital-year level in parentheses. CT, computed tomography; OLS, ordinary least squares; US, ultrasound.

**Fig. S1** Adjusted associations of non-resident status with the number of CT exams, with 95% confidence intervals; estimates control for triage code, having at least 1 US exam, weekend arrival, night arrival, and hospital, year, age, and motivation fixed effects; standard errors are clustered at hospital-year level. Adjusted differences in number of exams are reported for OLS panels; incidence rate ratios (IRR) in log scale are reported for Poisson and NB panels; odds ratios (OR) in log scale are reported for Logit panel. CT, computed tomography; ED, emergency department; NB, negative binomial; OLS, ordinary least squares; US, ultrasound.

**Fig. S2** Adjusted associations of non-resident status with the time-weighted imaging burden (in minutes), with 95% confidence intervals; estimates control for triage code, having at least 1 CT exam, having at least 1 US exam, weekend arrival, night arrival, and hospital, year, age, and motivation fixed effects; standard errors are clustered at hospital-year level. Adjusted differences in number of exams are reported for OLS panels; incidence rate ratios (IRR) in log scale are reported for Poisson and NB panels. CT, computed tomography; ED, emergency department; NB, negative binomial; OLS, ordinary least squares; US, ultrasound.
